# Axial Length and Prevalence of Myopia among Schoolchildren in the Equatorial Region of Brazil

**DOI:** 10.3390/jcm10010115

**Published:** 2020-12-31

**Authors:** Erisa Yotsukura, Hidemasa Torii, Hiroko Ozawa, Richard Yudi Hida, Tetsuro Shiraishi, Ivan Corso Teixeira, Yessa Vervloet Bertollo Lamego Rautha, Caio Felipe Moraes do Nascimento, Kiwako Mori, Miki Uchino, Toshihide Kurihara, Kazuno Negishi, Kazuo Tsubota

**Affiliations:** 1 Department of Ophthalmology, Keio University School of Medicine, 35 Shinanomachi, Shinjuku-ku, Tokyo 160-8582, Japan; erisa.tsutsui@gmail.com (E.Y.); hidemasatorii@yahoo.co.jp (H.T.); hiroko1011@gmail.com (H.O.); ryhida@gmail.com (R.Y.H.); morikiwako@gmail.com (K.M.); uchinomiki@yahoo.co.jp (M.U.); 2 Laboratory of Photobiology, Keio University School of Medicine, 35 Shinanomachi, Shinjuku-ku, Tokyo 160-8582, Japan; 3 Department of Ophthalmology, Universidade de Sao Paulo, Av. Dr. Enéas Carvalho de Aguiar, 255-Cerqueira César, São Paulo SP 05402-000, Brazil; yessa.rautha@yahoo.com.br; 4 Department of Ophthalmology, Universidade Federal de Sao Paulo, R. Botucatu, 822-Vila Clementino, São Paulo SP 04039-032, Brazil; 5 Department of Obstetrics and Gynecology, Keio University School of Medicine, 35 Shinanomachi, Shinjuku-ku, Tokyo 160-8582, Japan; shiraishi15.5@gmail.com; 6 Department of Ophthalmology, Santa Casa de Sao Paulo, Rua Dr. Cesario Mota Junior, 112-Vila Buarque, São Paulo SP 01221-010, Brazil; ivancorso@gmail.com (I.C.T.); caiofmn@gmail.com (C.F.M.d.N.); 7 Tsubota Laboratory, Inc., 304 Toshin Shinanomachi-Ekimae Bldg., 34 Shinanomachi Shinjuku-ku, Tokyo 160-0016, Japan

**Keywords:** myopia, refractive error, axial length, school children, light environment, violet light, illuminance

## Abstract

The prevalence of myopia is increasing globally, and the outdoor light environment is considered as a possible factor that can retard myopia. The aim of this study was to evaluate the prevalence of myopia and the light environment in Aracati, equatorial Brazil. We surveyed 421 children (421 right eyes; mean age, 10.6 years) and performed ocular examinations that included non-cycloplegic refraction and axial length (AL). Multiple regression analyses were performed to identify factors affecting myopia such as time spent outdoors and in near work. We measured illuminance and violet light irradiance in Aracati. The mean spherical equivalent (SE) and AL were −0.44 ± 1.38 diopters (D) and 22.98 ± 0.87 mm, respectively. The prevalence of myopia (SE ≤ −0.75 D) and high myopia (SE ≤ −6.0 D/AL ≥ 26.0 mm) was 20.4 and 1.4/0.48%, respectively. Multiple regression analyses showed that myopia was not associated with lifestyle factors. The average illuminance in Aracati was about 100,000 lux from morning to evening. The current results reflect the ALs and the prevalence of myopia among Brazilian schoolchildren. There is a possibility that the light environment in addition to other confounding factors including racial differences affects the ALs and refractive errors.

## 1. Introduction

Myopia, a common refractive error, mainly results from continuous increases in axial length (AL) [1]. The etiology of myopia is thought to be an interaction of genetic and environmental factors. However, the precise mechanism remains unclear. The prevalence of myopia has recently increased worldwide, especially in east Asia [2]. Currently, China has a high prevalence of myopia, while the prevalence rates have ranged from 20 to 80% for the previous 60 years; in other parts of the world such as the United States and Europe, the prevalence rates have doubled during the past 50 years [3]. We also recently reported that the prevalence of myopia among elementary school and junior high school children in Tokyo was 76.5 and 94.9%, respectively [4]. These rapid worldwide increases in myopia during a relatively short period must have resulted from changes in environmental factors and not genetic factors.

Outdoor activity is a factor that can suppress myopia onset [5,6,7,8,9] and progression [9] as reported by several studies including a meta-analysis. Spending more than 2 h daily outdoors is preferable [6,7,9]. The mechanism of the effects of outdoor activity remains unknown, but a possible reason is that outdoor activity can suppress myopia not only because of light illuminance [10,11] but also because of the short wavelength light, including violet light (360–400 nm wavelength) [12,13,14], that is abundant outdoors [12]. We reported that violet light might suppress myopia progression not only in chicks but also in children with mild myopia and adults with high myopia [12,13]. Based on those results, we hypothesized that the prevalence of myopia is lower in equatorial areas of Brazil, which have higher amounts of sunlight than other areas.

Numerous environmental factors have been associated with myopia progression in schoolchildren [15,16,17,18,19]; however, few studies have evaluated AL in schoolchildren using these factors, and those studies were mostly conducted in Asia [4,20]. To date, no reports have been published about AL and its associated factors in equatorial regions where the sunlight is intense, although a couple of studies have reported refractive data without AL among Brazilian schoolchildren [21,22,23]. Thus, using a questionnaire, we also evaluated the association between AL and environmental factors. We clarified the prevalence of myopia among schoolchildren in the equatorial region of Brazil by measuring refractive errors and ALs, and we investigated the light environment including the light illuminance and the violet light irradiance in Brazil.

## 2. Materials and Methods

### 2.1. Study Design and Participants

This cross-sectional study included 421 right eyes of 421 Brazilian schoolchildren (age range, 5–19 years; average age, 10.6 ± 2.9 (standard deviation) years; participation rate, 74.3%) who underwent a medical check-up that included the measurement of height and weight in the Professor Antonio Monteiro School in Pedregal, Aracati City, Ceará, Brazil, in August 2017. Aracati City is in the equatorial area of Brazil. We explained the study to the parents of the students. The students provided informed assent, and the parents provided written informed consent. All students who agreed to participate in this study underwent a medical examination. The inclusion criteria were children who could undergo an eye examination and had no previous ocular disease. The exclusion criteria were the presence of active ocular inflammation or systemic disease. In the questionnaire, we asked about a past history of systemic disease, and we evaluated active ocular inflammation during an ophthalmic medical examination. The Keio University School of Medicine Ethics Committee, and the Institutional Review Board and Ethics Committee of Penha Eye Institute approved this study. All the procedures involving human subjects were conducted in accordance with the tenets of the Declaration of Helsinki.

### 2.2. Measures

All participants underwent eye examinations that included measurement of refractive errors and AL. We also recorded the height, weight, and body mass index (BMI) of the participants. 

The refractive errors were measured in a non-cycloplegic state using an autorefractometer (VISUREF100, Carl Zeiss Meditec AG, Jena, Germany). We used the automatic fogging system to reduce the effects of accommodation when we measured the refraction. The ALs were measured by partial coherence interferometry biometry (IOLMaster^®^ 500, Carl Zeiss Meditec AG, Jena, Germany). Four trained ophthalmologists performed all the examinations, and the data were recorded. The device measured the AL five times and averaged the data. The spherical equivalent (SE) was defined as the spherical power plus half of the negative cylinder power. Myopia was defined as an SE of −0.75 diopters (D) or worse, and high myopia, as an SE ≤ −6.0 D or an AL ≥ 26.0 mm. Because the SE (Pearson correlation coefficient = 0.689, *p* < 0.001) and the AL (Pearson correlation coefficient = 0.959, *p* < 0.001) for the right and left eyes were similar for this study, only the results for the right eye are presented. All participants with any ocular disease were immediately treated and sent for further follow-up in Fortaleza City, Ceará, Brazil.

Illuminance was measured using the LX-1108 (MOTHERTOOL CO., Ltd., Ueda, Japan), and violet light irradiance was measured using VL-M-A1 (NEW OPTO, Ltd., Kawasaki, Japan). These data were collected outside in Aracati, Brazil (at 37° west longitude, 4° south latitude). All data were collected on sunny days at approximately 7:00, 10:00, 13:00, and 16:00 local time on 19 August 2017. 

### 2.3. Questionnaire

A questionnaire in Portuguese was administered before the visit. If the participants were too young to complete the questionnaire by themselves, Brazilian staff members interviewed them and completed the questionnaire with the help of their parents. Information on sociodemographic factors were collected, including age, sex, and family economic status. Using a questionnaire, we asked their parents about the average time of activity during one recent month. The children’s near work was assessed by asking (1) how many hours the child spent daily reading or studying for school assignments and for pleasure, using a computer, watching television, and playing with electronics, and (2) the distance when the child was reading. The children’s outdoor activity was measured by asking how long they spent in the sun daily while outdoors. The family economic status was assessed by asking for the entire family monthly income: less than BRL 1000, BRL 1000 to 3000, BRL 3000 to 5000, more than BRL 5000, or unknown. BRL 1 was equivalent to USD 0.18 on 19 August 2020. Parental myopia was assessed by asking the following question: “Is the child’s parent myopic?”. The response categories were “one parent”, “both parents”, “neither”, and “unknown”. Since some responded that the time spent sleeping was only 120 min/day during one recent month, we considered that the answers indicating that the children spent less than 300 min a day as unrealistic (*n* = 8) and treated them as if they were not a response.

### 2.4. Statistical Analysis

To evaluate the factors affecting the SE and AL, we performed multiple regression analyses (stepwise variable selection for regression). The Pearson correlation coefficient was used to assess simple correlations between the SE, AL, and several variables, such as age, sex (males = 0, females = 1), BMI, time spent outdoors, time spent in near work, family economic status, and parental myopia. Multiple regression analyses were performed, in which significant variables (*p* < 0.20) with simple correlations were compared. We confirmed that there was no multicollinearity. All *p* values were 2-sided. *p* < 0.05 was considered to be statistically significant. All statistical analyses were performed using the SPSS version 24.0 software (IBM, Chicago, IL, USA).

## 3. Results

### 3.1. Study Population

**Figure 1:**
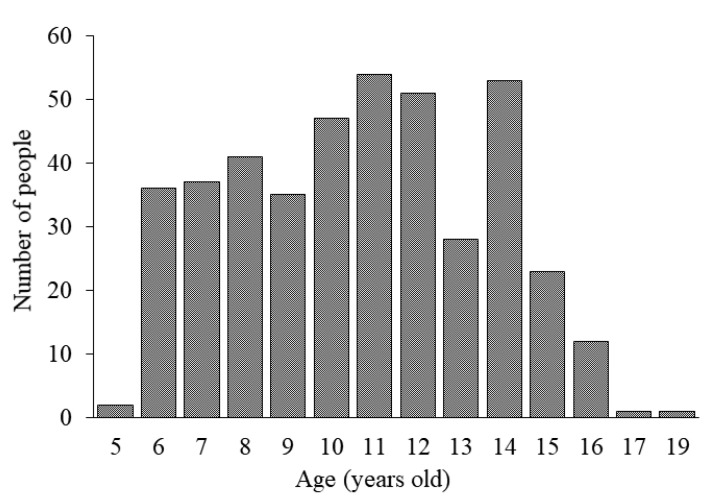
The distribution of subjects at each age: The ages of the participants in this study were mainly distributed from 6 to 16 years. The average age was 10.6 years.

**Figure 2:**
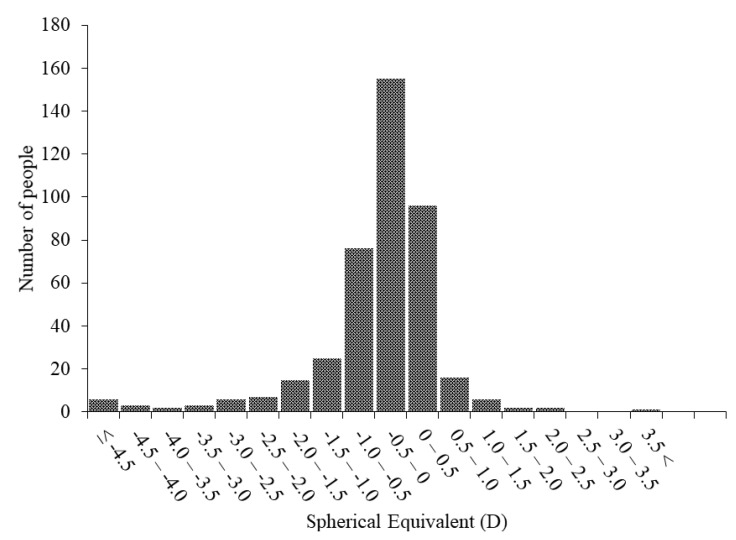
The distribution of SE. The participants in this study were primarily emmetropic (average, −0.44 diopters). Abbreviations: SE, spherical equivalent; D, diopters.

**Figure 3:**
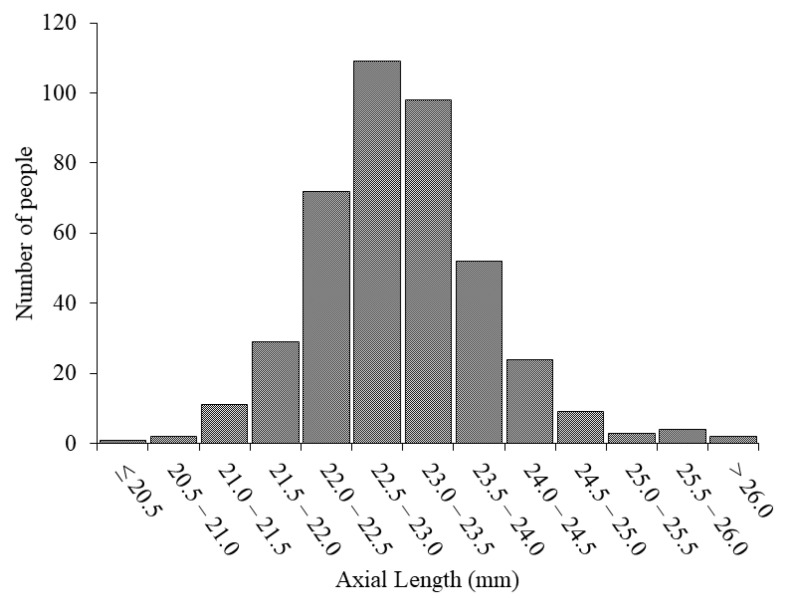
The distribution of the axial lengths (ALs). The ALs of the participants in this study were primarily in the 21.0 to 24.5 mm range (average, 22.98 mm).

Table 1 shows the characteristics of the current study population and results of the questionnaire. The mean SE was −0.44 ± 1.38 D, and the mean AL was 22.98 ± 0.87 mm for all the participants. The average time spent outdoors was 155.9 ± 104.3 min/day, and the average time spent doing near work was 516.4 ± 248.3 min/day. Figure 1 shows the distribution of the subjects by age. The distributions of the SE and AL are shown in Figure 2 and Figure 3, respectively.

The prevalence of myopia (SE ≤ −0.75 D) was 20.4%, and the prevalence of high myopia (SE ≤ −6.0 D) was 1.4% among all the students. Based on the AL, the prevalence of high myopia (AL ≥ 26.0 mm) was 0.48% among all the students.

### 3.2. Illuminance and Violet Light Irradiance

The illuminance and violet light (360–400 nm) irradiance values in Aracati and Tokyo are shown in Figure S1 (The illuminance and violet light irradiance in Tokyo and Aracati). The illuminance (lux) at 7:00, 10:00, 13:00, and 16:00 local time in Aracati/Tokyo was 92,000/76,000, 103,000/93,300, 107,500/106,000, and 80,000/54,000, respectively (Figure S1A). The violet light irradiance (µW/cm^2^) at 7:00, 10:00, 13:00, and 16:00 local time in Aracati/Tokyo was 1650/1120, 2300/2000, 2320/1950, and 1030/468, respectively (Figure S1B). 

### 3.3. Factors Affecting SE and AL According to Simple Correlation and Multiple Regression Analyses

Table 2 shows the results of simple correlation and multiple regression analyses between the SE and the variables. The results indicate that myopia is associated with older age (coefficient = −0.170; *p* < 0.001). Based on the results of the multiple regression analyses between the AL and the variables, the AL was associated with older age (*p* < 0.001), male sex (*p* < 0.001), and higher BMI (*p* = 0.01) (Table 3). Lifestyle factors were included (*n* = 114), and less sleep time was associated with a longer AL according to simple correlation (coefficient = −0.184; *p* = 0.025). The results of multiple regression analysis show that the AL was significantly associated with older age and male sex (coefficient = 0.119 and −0.405, respectively; *p* = 0.001 and 0.002, respectively).

## 4. Discussion

**Figure 4:**
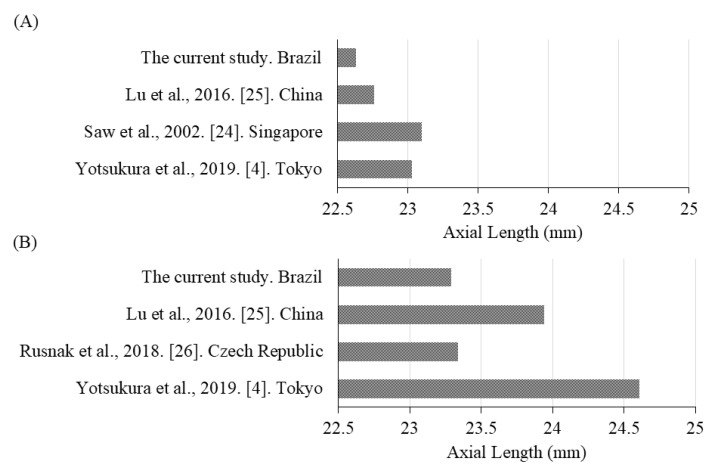
Comparison of the axial lengths (ALs) of (**A**) 7 and (**B**) 12 year-old schoolchildren in the current study with those from previous studies. Compared with some previous studies, the ALs of both the 7 and 12 year-old schoolchildren in the current study were the shortest.

The current results show that the mean refractive error among the Brazilian schoolchildren was −0.44 ± 1.38 D, the myopia prevalence in Aracati was 20.4%, and the prevalence of high myopia was 1.4%. In the current study, the mean AL was 22.98 ± 0.87 mm, and based on the AL, the prevalence of high myopia (AL ≥ 26.0 mm) was 0.48%. These results show a possibility that children in an equatorial area of Brazil might have shorter ALs than those in other studies [4,24,25,26] (Figure 4). A search of PubMed was undertaken for articles including the search terms “axial length”, “children”, and “myopia”, and we chose the references in which the age was the same as in our study (7 and 12 years old) and the studies that included more than 300 participants. In the current study, the mean ALs of the schoolchildren aged 7 and 12 years were 22.63 and 23.29 mm, respectively, which were the shortest among these studies [4,24,25,26] (Figure 4A,B). Possible reasons why the mean ALs of the schoolchildren aged 7 and 12 years in the current study were the shortest among these studies [4,24,25,26] are race and extended time of outdoor activity. Unfortunately, we could not obtain accurate information about the participants’ races. The mechanism of outdoor activity’s effect remains unknown, but a possible reason is the strong light environment including light illuminance [10,11] and the short wavelength of the light [12,13,14] that is abundant outdoors. We measured them only during 1 day, but there were no such published data, and we used them as Supplementary Data.

We also compared our data with a previous study from Brazil to eliminate the racial differences. According to a previous study [27], which reported the ALs among Brazilian schoolchildren in Campinas (at 46° west longitude, 23° south latitude), the mean ALs were 22.5, 23.0, and 23.2 mm among the children aged 6, 10, and 14 years, respectively. In the current study, the mean ALs were 22.5, 23.0, and 23.0 mm (almost plateaued) among the children aged 6, 10, and 14 years, respectively. The mean ALs were the same for those aged 6 and 10 years, though we focused on the ALs of those aged 14 years. In Campinas, the mean AL for those aged 14 years was 23.2 mm; on the other hand, it was 23.0 mm in Aracati. The results indicate that the AL might plateau in Aracati at around 10 years because the mean ALs were same for those aged 10 and 14 years. These results show that the mean ALs of the adolescent children in Aracati plateaued and were shorter than those in Campinas. These results indicate that the ALs of children near the equatorial region where the sun light is intense in the same country might be short without factors such as the time spent outdoors and genetic backgrounds.

Several studies have suggested that increased time spent in near work is a possible risk factor for myopia [18,28,29,30,31,32]. Indeed, the time spent doing near work has been increasing recently among students [33,34]. When we evaluated the time spent doing near work in Aracati, the mean time was 516.4 min/day. By contrast, the time spent doing near work in Tokyo was 274.0 min/day [4]. However, in our previous study, which reported the prevalence of myopia among schoolchildren in Tokyo, we asked parents about the average time of activity excluding school time; therefore, it is possible that the time spent doing near work is almost the same in Aracati and Tokyo. 

Outdoor activity is a well-known factor that suppresses myopia onset [7,35,36,37,38,39,40], and some clinical studies [41,42,43] have reported that outdoor activity may suppress myopia progression. In the current study, the mean time spent outdoors among Brazilian children was 155.9 min/day. However, the mean time spent outdoors among Japanese elementary school children was 78.3 min/day [4], which was about half of that in Aracati. Some studies have reported that spending more than 2 h daily outdoors can suppress myopia [6,7]. According to the results of the current study, the low prevalence of myopia in Aracati may be associated with the extended amount of time engaged in outdoor activities despite the long periods spent doing near work. However, the time spent engaged in outdoor activities was not significantly associated with myopia in the current study. We consider this because the mean time spent outdoors among children in Aracati was over 2 h daily (155.9 min daily). The effect of decreasing the risk of incident myopia almost plateaued under that condition, i.e., over 2 h daily of outdoor activity, according to a systematic review of the effects of outdoor activity against myopia [8]. For that reason, the time spent outdoors was not significantly associated with myopia in the current study.

The outdoor light environment, including the illuminance and wavelength, is now attracting attention in attempts to retard myopia [14]. High illuminance levels have been effective against myopia in some animal experiments [11,44,45]. Furthermore, we recently found that 360 to 400 nm violet light, which is abundant in sunlight, suppressed myopia progression in both chicks and humans [12], i.e., not only in children with mild myopia but also in adults with high myopia [13]. Therefore, we measured the illuminance and violet light irradiance in the current study. Although we did not compare the illuminance and irradiance of violet light globally and annually, a website titled “World Weather Online” [46] shows the ultraviolet index (UVI), which was developed by the World Health Organization and is a measure of UV radiation levels. The calculated annual average UVI in 2017 was 6.75 in Aracati, 6.92 in Singapore, and 4.25 in Tokyo [46]. Although the UVIs in Singapore and Aracati were almost the same, the prevalence of myopia in Singapore was very high [47,48,49] because of differences in the time spent outdoors and genetic backgrounds between Singapore and Aracati. A previous study [50] showed that the average time spent outdoors among Singaporean children was only 61 min/day, although the time spent outdoors in Aracati was 155.9 min/day in the current study. Even though genetic backgrounds affected the rate of myopia prevalence, the time spent outdoors in Aracati, which was almost 2.5 times more than that in Singapore, may also affect ALs and refractive errors. Therefore, environmental factors including both the time spent outdoors and light environment are crucial factors for the prevalence of myopia.

The current multiple regression analysis results indicated that AL was significantly associated with older age and male sex. In the current study, there was no significant relationship between myopia and lifestyle factors. Regarding the time spent sleeping, the average time among the Brazilian children was 507.8 min daily in the current study, and the time among Japanese school children was 525.2 min daily, which we reported previously [4]. Thus, the time spent sleeping among Brazilian students was less than that of Japanese students by about 20 min daily. A previous study reported that high myopia was significantly correlated with short sleep duration in Japan [51], although the time spent sleeping among Brazilian children was shorter; nevertheless, their ALs were shorter than those of the Japanese school children. These investigations imply that lifestyle factors are not associated with myopia in the equatorial area of Brazil because of the light environment including the illuminance, annual daylight hours, and violet light irradiance. Thus, the current study indicates that the intensity of the sunlight may play an important role in the low prevalence of myopia and short AL among the Brazilian schoolchildren in equatorial regions.

The current study had some limitations. First, we did not use cycloplegia. The non-cycloplegic refraction was a big limitation of this study, especially for children because the non-cycloplegic refraction masked the real refraction as a result of their accommodative ability. Considering that, the prevalence of myopia may have been overestimated. We also might have overestimated the AL because a previous study reported that the AL was 0.009 mm shorter after cycloplegia in children whose mean age was 9.20 years [52]. Since the difference was small, the ALs in Brazilian schoolchildren tended to be short, and measuring the AL was the strength of the current study. Second, there may have been some recall bias and misunderstanding of the questionnaire. Third, we could not obtain accurate information about the participants’ races because the percentage of mixed-race individuals was about 43%, according to The Brazilian Institute of Geography and Statistics. It may be better to choose another place in Brazil where the sunlight is less intense because the races in Brazil and Japan differ. Fourth, over half of the students did not completely answer the questionnaire about their lifestyle, and we could perform multiple regression analysis about lifestyle for only 114 participants. We considered that the lack of association between lifestyle factors and myopia could be because of a lack of power. Finally, we measured only the violet light irradiance because we focused on violet light in the current study. We should have measured not only violet light but also other spectral components of light.

## 5. Conclusions

In summary, the current results reflect the ALs and the prevalence of myopia among Brazilian schoolchildren. There is a possibility that the light environment in addition to other confounding factors including racial differences affects their ALs and refractive errors, and further studies are needed in the future.

## 6. Patents

Outside the submitted work, but related to myopia prevention, H.T., T.K., and K.T. have been applying internationally for two patents, WO 2015/186723 and WO 2017/094886. The former has been registered in Japan, the U.S., and China, and the latter, in Japan, the U.K., France, Germany, Italy, Hong Kong, and Singapore.

## Figures and Tables

**Table 1:** Characteristics of the current study population and results of the questionnaire.

	Number of Cases	Mean ± Standard Deviation (Range)
Age (years)		10.6 ± 2.9 (5.0–19.0)
Number of students	421	
Males	212	
Females	209	
Height (cm)	417	144.5 ± 15.6 (110.0–179.0)
Weight (kg)	416	40.4 ± 13.9 (16.7–91.2)
BMI (kg/m^2^)	416	18.8 ± 3.6 (12.1–31.9)
SE (D)	421	−0.44 ± 1.38 (−12.63–7.25)
AL (mm)	416	22.98 ± 0.87 (20.16–26.90)
Time spent outdoors (min/day)	123	155.9 ± 104.3 (9.3–540.0)
Time spent in near work (min/day)	121	516.4 ± 248.3 (60.0–1096.5)
Time spent sleeping (min/day)	113	507.8 ± 100.7 (300.0–875.0)
Family economic status		
Less than BRL 1000	42	
BRL 1000 to 3000	19	
BRL 3000 to 5000	5	
More than BRL 5000	5	
Unknown	171	
Number of parents with myopia		
0	158	
1	38	
2	6	
Unknown	219	

Abbreviations: SE, spherical equivalent; AL, axial length; BMI, body mass index; D, diopter. BRL 1 was equivalent to USD 0.18 on 19 August 2020.

**Table 2:** Results of simple correlation and multiple regression analysis between SE and variables (*n* = 416).

	Univariate Analysis		Multivariate Analysis		
Variable	Correlation Coefficient	*p* Value	Coefficient	Standard Error	*p* Value
Age (years)	−0.170	<0.001 *	−0.082	0.023	<0.001 *
Sex (males = 0, females = 1)	−0.041	0.201	N/A	N/A	≥0.05
BMI	−0.075	0.062	N/A	N/A	≥0.05

* Significant correlation according to the Pearson correlation test; Abbreviations: SE, spherical equivalent; BMI, body mass index; N/A, not applicable.

**Table 3:** Results of simple correlation and multiple regression analysis between AL and variables (*n* = 414).

	Univariate Analysis		Multivariate Analysis		
Variable	Correlation Coefficient	*p* Value	Coefficient	Standard Error	*p* Value
Age (years)	0.326	<0.001 *	0.079	0.015	<0.001 *
Sex (males = 0, females = 1)	−0.267	<0.001 *	−0.438	0.078	<0.001 *
BMI	0.248	<0.001 *	0.031	0.012	0.01 *

* Significant correlation according to the Pearson correlation test; Abbreviations: AL, axial length; BMI, body mass index.

## Data Availability

The data presented in this study are available on request from the corresponding author. The data is stored, and it will be discarded as the approved document by Ethics Committee.

## Supplementary Materials

The following are available online at https://www.mdpi.com/2077-0383/10/1/115/s1, Figure S1: The illuminance and violet light irradiance in Tokyo and Aracati.
