# Supplementary material for: Axial Length and Prevalence of Myopia among Schoolchildren in the Equatorial Region of Brazil
Source: J Clin Med. 2020 Dec 31;10(1):115. doi: 10.3390/jcm10010115 (PMC7794886; doi:10.3390/jcm10010115)
Supplement: Supplementary file 1 [file jcm-10-00115-s001.pdf]

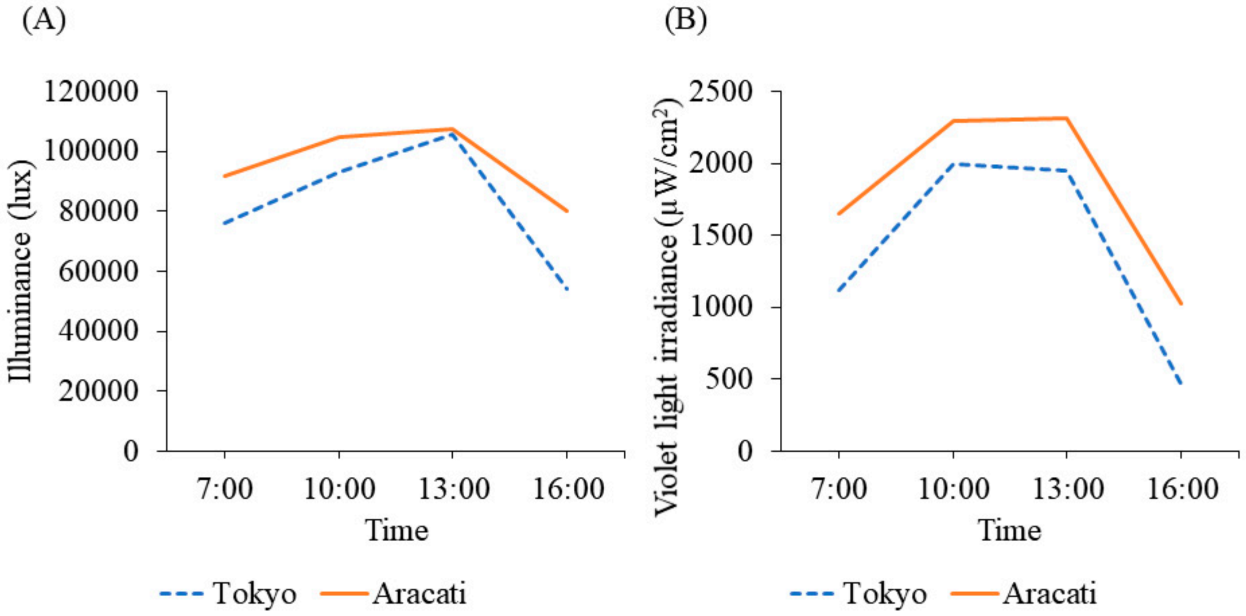

Figure 1. The illuminance and violet light irradiance in Tokyo and Aracati. The illuminance and violet light irradiance in Aracati and Tokyo are shown in Figure S1 (**A**) and (**B**). (**A**) The average illuminance during the day in Aracati is almost the same as in Tokyo. (**B**) The average violet light irra-diance during the day in Aracati is higher than in Tokyo.
